# HOSPITALIZATIONS OF OLDER INCARCERATED ADULTS: HOW ARE THEY DIFFERENT?

**DOI:** 10.1093/geroni/igad104.1605

**Published:** 2023-12-21

**Authors:** Farah Kaiksow

**Affiliations:** University of Wisconsin School of Medicine and Public Health, Madison, Wisconsin, United States

## Abstract

By 2030, over one-third of the US prison population will be ≥55 years old. In addition to their biological age, incarcerated patients are at increased risk of geriatric conditions due to accelerated aging. As these individuals age, so will their need for community hospitalizations, which already account for 20% ($1.5 billion annually) of state correctional health spending. To date, there has been very limited data on these hospitalizations. This descriptive study provides the largest amount of information yet, comparing 10 years of data on male incarcerated patients (N=4,401 admissions) to a cohort of nonincarcerated males (N=22,150) from a tertiary care center. Incarcerated patients were more likely to be ≤55 than non-incarcerated (53.9% vs. 20.7%) and have shorter lengths of stay (4.7 vs. 5.8 days). The most common diagnosis for both groups was cardiac-related (ICD-10 Chapter 9), while mental health diagnoses (ICD-10 Chapter 5) were the second most common for incarcerated patients but not in the top five for non-incarcerated. Incarcerated patients had higher Charlson Comorbidity Index scores, with incarcerated patients having higher scores beginning at age 50 than non-incarcerated ≥80. Surprisingly, the one-year mortality rate for incarcerated patients was almost half that for non-incarcerated (56/1,000 vs. 101/1,000); one possible explanation for this disparity is that the community health system data on incarcerated patients may be incomplete. This study supports the theory of accelerated aging among incarcerated individuals and highlights the need for improved data on which to base policy changes to improve the care of this vulnerable population.

